# Hyperbaric Medicine in Pediatrics — reality of a Portuguese reference center

**DOI:** 10.1590/1984-0462/2025/43/2023230

**Published:** 2024-07-08

**Authors:** Catarina Freitas, Luís Salazar, Sílvia Duarte-Costa, Catarina Fraga, Sara Monteiro, Óscar Camacho

**Affiliations:** a Unidade Local de Saúde de Matosinhos – Porto, Portugal.; b Centro Hospitalar Universitário do Porto – Porto, Portugal.

**Keywords:** Pediatrics, Hyperbaric oxygen therapy, Pediatrics emergency, Carboxyhemoglobin, Pediatria, Oxigenoterapia hiperbárica, Emergência pediátrica, Carboxiemoglobina

## Abstract

**Objective:**

To identify and characterize the population of Pediatric patients referred to our hyperbaric oxygen therapy center.

**Methods:**

Retrospective and observational study, including pediatric patients treated with hyperbaric oxygen therapy, from 2006 to 2021, at the hyperbaric medicine reference center in the north of Portugal. Variables of interest were extracted from electronic medical records.

**Results:**

Our study included 134 patients. The most frequent reasons for referral were carbon monoxide poisoning (n=59) and sudden sensorineural hearing loss (n=41). In 75 cases (56%), treatment was initiated in an urgent context. Symptom presentation at Emergency Department varied among patients, the most frequent being headache and nausea/vomiting. Concerning carbon monoxide poisoning, the most common sources were water heater, fireplace/brazier, and boiler. Regarding adverse effects, it was identified one case of intoxication by oxygen and four cases of middle ear barotrauma.

**Conclusions:**

The most frequent cause for referral was carbon monoxide poisoning. All patients evolved favorably, with few side effects being reported, emphasizing the safety of this therapy. While most pediatricians may not be aware of the potential benefits arising with hyperbaric oxygen therapy, it is of upmost importance to promote them, so that this technique is increasingly implemented.

## RESUMO

**Objetivo:**

Identificar e caracterizar a população de casos pediátricos encaminhados para o nosso centro de oxigenoterapia hiperbárica.

**Métodos:**

Estudo retrospetivo e observacional, que incluiu doentes pediátricos tratados com oxigenoterapia hiperbárica, de 2006 a 2021, no centro de referência de medicina hiperbárica do norte de Portugal. As variáveis de interesse foram extraídas dos processos clínicos eletrônicos.

**Resultados:**

O nosso estudo incluiu 134 casos. Os motivos de encaminhamento mais frequentes foram intoxicação por monóxido de carbono (n=59) e surdez súbita neurossensorial (n=41). Em 75 casos (56%) o tratamento foi iniciado em contexto de urgência. Os sintomas de apresentação à admissão variaram entre os diferentes casos, sendo os mais frequentes cefaleias e náuseas/vômitos. No que diz respeito à intoxicação por monóxido de carbono, as fontes mais comuns foram o aquecedor, lareira/braseiro e caldeira. Com relação aos efeitos adversos, foram identificados um caso de intoxicação por oxigênio e quatro casos de barotrauma do ouvido médio.

**Conclusões:**

A causa mais frequente de encaminhamento foi a intoxicação por monóxido de carbono. Todos os pacientes evoluíram favoravelmente e foram registrados poucos efeitos adversos, o que enfatiza a segurança desta terapia. Uma vez que a maioria dos pediatras pode não estar informada sobre os potenciais benefícios da oxigenoterapia hiperbárica, é de extrema importância promovê-los para que esta técnica seja cada vez mais implementada.

## INTRODUCTION

Hyperbaric oxygen therapy (HBOT), an old therapeutic approach, has been more frequently used in the pediatric population. According to scientific hyperbaric societies, HBOT is a medical treatment based on breathing 100% oxygen in hyperbaric chambers at pressures higher than 1.4 atmosphere absolute pressure for clinical effect. Treatment is performed in monoplace or multiplace chambers at pressures between 2.0 and 2.8 atmospheres for 90–120 minutes. Oxygen is inhaled through a mask, hood, or endotracheal tube, or from ambient air if the chamber is pressurized with 100% oxygen. These high doses of oxygen have considerable beneficial biochemical, cellular, and physiological effects.^1–3^

One of the therapeutic effects of hyperbaric oxygen results from the mechanical impact of increased pressure in the gas-containing body environment and the physiological effects induced by enhancing oxygen delivery. Hypoxia causes an elevation in oxidative stress, leading to the production of reactive free radicals of oxygen and nitrogen. These free radicals, highly toxic to cells, cause damages that trigger cellular death and apoptosis.

It should be noted that inhaling high levels of oxygen has a negligible effect on the total oxygen content of hemoglobin. However, HBOT increases the amount of oxygen dissolved in the plasma, thus making it available for use at the tissue level. This phenomenon is of utmost importance when tissue oxygenation is compromised.

Oxygen also has an antimicrobial effect, particularly for anaerobic bacteria. For normal phagocytosis and oxidative processes to occur, an oxygen pressure of at least 30 mmHg is necessary. In damaged tissues, oxygen is available in a much lower quantity, so increasing it through HBOT can restore the function of defense cells, allowing adequate antimicrobial action.

In addition, oxygen has a vasoconstrictor effect, meaning it has the potential of reducing tissue edema. In a hypoxic environment, wound healing is compromised by local infection and a decrease in fibroblast proliferation, collagen synthesis, and capillary angiogenesis. Thus, hyperbaric oxygen has been shown to restructure the favorable cellular environment for wound healing along with its antimicrobial effect.^4^

HBOT plays a role in addressing hypoxic conditions by enhancing oxygen delivery, promoting antimicrobial activity, and mitigating the effects mediated by hypoxia-inducible factors. Therefore, several pathologies and clinical situations benefit from this treatment. The European Committee for Hyperbaric Medicine aims to improve the quality and safety of Hyperbaric Medicine, making recommendations on which clinical situations are indicated for this type of treatment, grouped according to recommendation level.^5^

Regarding the pediatric population, there are still few studies about the specific conditions that can benefit from HBOT. However, there is growing evidence supporting the effectiveness of treatment in specific pathologies/situations, such as carbon monoxide (CO) poisoning, soft tissue necrosis, bone necrosis, burns, and after skin transplants.

The multiplace hyperbaric chamber in Hospital Pedro Hispano — the reference center for this therapy in northern Portugal — has capacity for 16 seated patients. It is a level-3 chamber, that is, with the capacity to monitor respiratory and hemodynamic parameters, institute invasive ventilation, and treat critical patients. Patients proposed for routine treatment undergo a prior evaluation to validate the therapeutic indication for HBOT, rule out any contraindication for treatment, submit to any complementary diagnostic exam that is identified as necessary, and sign the informed consent for treatment, which is carried out by the patient or a legal substitute. In the case of urgent patients, after confirming the diagnosis for treatment with HBOT, they undergo a primary and secondary assessment, are clinically stabilized, and are monitored depending on the severity.

During routine and urgent treatments, all patients are always accompanied by a nurse and/or doctor. In the case of pediatric patients, when possible, they are also accompanied by a family member.

Our purpose was to identify the different pathologies referred for HBOT in pediatric age, characterizing the reality of the Hospital Pedro Hispano as well as evaluate the effectiveness and complications of this therapy.

## METHOD

We conducted a retrospective and observational study of pediatric patients referred for treatment with HBOT at a regional hyperbaric referral center, in northern Portugal, between 2006 and 2021. Patient records were identified by the International Classification of Diseases coding as well as the indications of a consult to the hyperbaric medicine team. The exclusion criteria were age over 18 years and insufficient/incomplete clinical records — 18 cases were excluded. The ethics committee for health at Local Health Unit (ULS) Matosinhos decided, unanimously, that there was nothing to oppose the performance of this study. Variables of interest were extracted from electronic medical records. Data were collected on demographic characteristics (identity information, age, gender), presenting symptoms, the reason for referral, number of sessions performed, evolution and complications arising from treatment. Data extraction was completed by a team of five medical doctors, all with prior exposure to electronic medical records, and thereafter a database was created using Microsoft Access. Data analysis began after the database was completed and was carried out by a member of the research team who was also involved in data extraction. Descriptive analyses were conducted to examine patient variables and statistical analysis was subsequently performed using Statistical Package for Social Sciences (SPSS^®^), version 21.0 software.

## RESULTS

From 2006 to 2021, 134 children were treated with HBOT at Hospital Pedro Hispano. Of these, 77 (57.5%) were females. Age ranged 1–18 years, with a median of 14 years. In 75 cases, treatment was initiated in an urgent context (referred to the emergency services). The reasons for referral for HBOT treatment are illustrated in Table 1. The two most frequent indications were CO poisoning (n=59) and sudden sensorineural hearing loss (n=41).

**Table 1:** Reasons for referral for treatment with hyperbaric oxygen therapy in Hospital Pedro Hispano.

Indications	n	Age, years mean (range)	Gender Male/Female	No. sessions mean (range)
Carbon monoxide poisoning	59	10.9 (1–18)	18/41	1.7 (1–3)
Sudden sensorineural hearing loss	41	14.8 (7–18)	20/21	19.8 (2–30)
Soft tissue necrosis and wound healing	11	13.4 (8–18)	7/4	20.7 (4–57)
Hemorrhagic radiation-induced cystitis	12	10.3 (1–18)	5/7	12.7 (1–28)
Sports injury	6	16.3 (15–18)	5/1	9.5 (4–15)
Chronic refractory osteomyelitis	5	16.8 (15–18)	2/3	39.6 (18–60)

Regarding CO poisoning, a total of 59 pediatric patients were treated at our center, 69.5% (n=41) of them were female. All cases were referred to the Emergency Department, particularly in the winter months (November to April). Median age was 11 years (range 1–18) and mean exposure time was 5.6 hours (range 0.5–16). Median blood carboxyhemoglobin (COHb) level at the time of presentation was 20.8 (range 7.1–40.2) and the average lactate value was 3.2 mmol/L (range 2.9–6.3). As shown in Table 2, the source of CO in these children was water heater in 23 (38.9%) cases, fireplace/brazier in 19 (32.2%), boiler in 11 (18.6%), and unknown in six cases. Symptom presentation at the Emergency Department varied among patients, as revealed in Table 3, the most frequent being headache (n=42) and nausea/vomiting (n=34). None presented coma at the Emergency Department.

**Table 2:** Source of carbon monoxide, exposure duration, and carboxyhemoglobin level at presentation in Emergency Department, relative to patients’ age.

Source of carbon monoxide	n	Age median (IQR), years	Duration of exposure median (IQR), hours	COHb level at presentation, median (IQR), %
Water heater	23	11.2 (1–18)	2.8 (0.5–7)	20.1 (7.1–33.1)
Fireplace/brazier	19	10.4 (5–14)	7.8 (2–14)	28.8 (8.4–40.2)
Boiler	11	14.7 (3–17)	11 (2–16)	18.9 (11.7–27.5)
Unknown	6	9.3 (2–18)	9.5 (4–15)	15.7 (13.5–17.6)

IQR: interquartile range; COHb: carboxyhemoglobin.

**Table 3:** Symptoms at Emergency Department.

Signs/symptoms in the Emergency Department*	n (%)
Headache	42 (71.2)
Nausea/vomiting	34 (57.6)
Transient loss of consciousness	21 (35.6)
Tachycardia	17 (28.8)
Dizziness	14 (23.7)
Asthenia	9 (15.3)

* Multiple pediatric patients had more than one presenting symptom.

After just one HBOT session, 27 children recovered. Nonetheless, most required a second treatment session 12 hours after the first. Specifically, 30 children needed two sessions and only two required three sessions.

A total of 41 pediatric patients were referred to our center because of sudden hearing loss. Median age was 14.8 years (range 7–18), and 51.2% (n=21) were female. All cases of deafness had already unsuccessfully undergone first-line treatments, so they were referred for treatment with HBOT (up to three weeks after onset of disease) with improvement in all of them. The average number of sessions was 19.8 (range 2–52). All patients were referred after examination by Otorhinolaryngology and subsequent confirmation of severe hearing impairment or deafness through an audiogram. The cause of deafness remained unidentified in the majority of cases (78%; n=32).

We found five patients referred to HBOT for chronic wounds: four patients for skin graft feasibility (after burns) and two for gas gangrene. The median number of sessions in these cases is represented in Table 4. In the four cases of skin graft viability after burns, the temporal median reference was approximately six months after conventional treatment had failed, characterized by attempts at healing through secondary intention and previous unsuccessful grafting. In the two cases of gas gangrene, HBOT was used early to control the infectious focus and then as an adjuvant measure to promote healing and enable skin grafts.

**Table 4:** Average number of sessions by type of reference.

Type of reference	n	Number of sessions median (IQR)
Chronic wound	5	32.5 (6–57)
Skin graft feasibility	4	14.8 (4–23)
Gas gangrene	2	8.5 (7–10)

IQR: interquartile range.

In 12 cases, the reason for referral for treatment with HBOT was hemorrhagic cystitis, after radiotherapy. Median age was 10.3 years (range 1–18). In our series, 72.4% of patients had completely resolved hematuria, and the remaining had a decrease in intensity and frequency.

Five patients with osteomyelitis, who showed no improvement with systemic antibiotic treatment were referred for HBOT. Median age was 16.8 years (range 15–18). The number of sessions varied between 18 and 60, with an average of 39.6 sessions. All cases showed resolution of the condition after undergoing HBOT.

Six patients were referred to HBOT for sports injuries and their median age was 16.3 years (range 15–18). Regarding the type of injury, four cases involved knee trauma (three posterior cruciate ligament ruptures and one anterior cruciate ligament rupture), while in the other two, the lesions were foot stress fractures. The four injuries occurred during football practice, one during tennis practice (posterior cruciate ligament rupture), and another happened in artistic gymnastics (foot stress fracture); all were referred after orthopedic assessment. Sports activities had to be discontinued during treatment in all cases, except gymnastics. Resolution of complaints was observed in all patients, after an average of 9.5 sessions (range 8–12) of HBOT. In cases of football injuries, after three months they were already carrying out conditioning training.

Regarding adverse effects, one case of toxic effect of oxygen presenting irritability and nausea was identified in our study, which was reversed after stopping treatment. The four cases of middle ear barotrauma, median age 6.5 years (range 5–7), recovered only with medical treatment. Paracentesis of the tympanic membrane before treatment was performed in five cases (all <3 years old children), in order to prevent this adverse effect.

## DISCUSSION

In our study, a favorable evolution was observed in all children, without any association with side effects, illustrating the safety of HBOT and emphasizing the importance of correct and timely referrals.

CO emerges from the incomplete combustion of carbon-included fuels. Accidental CO poisoning, due to malfunctioning heating systems or water heaters, is very common during winter, particularly in northern Portugal, for social, economic, and climatic reasons. Clinical signs of CO poisoning range from flu-like symptoms (such as headache, nausea, and vertigo) in mild cases, to severe cardiovascular and neuropsychological symptoms. In severe instances, these symptoms can progress to coma and even death. Neuropsychological manifestations include impairment in cognitive function, mood disorders, and irritability.^6^ The signs and symptoms exhibited by children may differ from those observed in adults and can be difficult to identify. They include irritability, seizure, focal neurologic deficit, and altered mental status. Consequently, it is crucial to maintain a high level of suspicion for CO poisoning when a child is taken to the emergency room from an environment where exposure could have occurred.^7,8^ In our sample, the most common symptoms were headache, nausea/vomiting, and transient loss of consciousness. CO has an affinity for hemoglobin 200 times greater than oxygen. Even at low inhalation levels, when CO binds to hemoglobin (forming COHb), it causes a leftward shift in the oxygen dissociation curve, resulting in reduced oxygen delivery to tissues. Thus, at the cellular level, the formation of oxygen free radicals occurs, leading to cellular damage caused by lipid peroxidation, which can ultimately contribute to brain damage. Lactate resulting from anaerobic glycolysis is an important marker of tissue hypoxia. In children admitted due to CO poisoning, blood lactate levels may give more accurate information about the degree of consciousness loss and convulsions. Therefore, lactate level could be considered as a measure of severe poisoning and may help decide on hyperbaric oxygen treatment.^9^ In our sample, the average lactate value was 3.2 mmol/L (2.9–6.3). All cases were referred for HBOT by the Emergency Department and treatment started on time. According to the literature, adverse cognitive outcomes (including issues with concentration and memory) may manifest shortly following exposure and endure, or might have a delayed onset. This may be difficult to assess at pediatric age. The research suggests that cognitive consequences persisting for a month or longer seem to be present in 25–50% of individuals who experienced loss of consciousness or had COHb levels exceeding 25%.^10^

Therefore, under these conditions, referral for HBOT is essential. In our sample, 21 children presented loss of consciousness, which was an immediate indication for HBOT, followed by a period of surveillance. Due to their underdeveloped nervous system, children may face specific susceptibility to what is described as "delayed neurologic sequelae" (DNS). This includes memory difficulties, chronic headaches, and even decline in academic performance. The research on CO poisoning in children is limited, with even fewer studies specifically focusing on clinical manifestations.^7,11^ Therefore, although there are no defined criteria to determine DNS risks, timely diagnosis in the Emergency Department could lead to enhanced quality of care, particularly in terms of preventing adverse events related to the central nervous system.^12^

Sudden sensorineural hearing loss is a significant clinical emergency characterized by an unexplained hearing reduction of at least 30 decibels in three or more consecutive frequencies, manifesting within a three-day period. Hearing loss can be induced by retrocochlear causes, such as stroke or acoustic neuroma, and in these patients, the treatment is directed to the underlying cause.^13–15^ However, in most cases, as in our study, the etiology remains unknown (idiopathic hearing loss) and may be due to viral infections, vascular occlusions, or immune-associated mechanisms.^13^ In this situation, corticosteroid therapy is given, as it is the only mode of treatment with proven efficacy at present. Nonetheless, literature supports that HBOT can be employed as the primary treatment approach alongside corticosteroids or as a secondary option for individuals who do not respond to corticosteroids.^13–15^ It is also described that the combination of oral steroid therapy and HBOT is most effective when compared to oral steroid therapy, intratympanic steroid therapy, and HBOT alone.^15,16^ The purpose of HBOT is to increase partial oxygen pressure in blood and inner-ear fluids. A recent guideline suggests administering corticosteroid treatment along with HBOT within two weeks following the onset of sudden sensorineural hearing loss.^17^

In our sample, all cases of deafness had already undergone first-line treatment without any benefit; thus, they were referred for HBOT (up to three weeks after onset of disease) with improvement in all cases. This highlights the effectiveness of salvage HBOT for sudden sensorineural hearing loss, which is performed after three weeks of disease onset.

Successful wound healing relies on the presence of oxygen, as it is essential for heightened reparative activities like cell growth, formation of new blood vessels (angiogenesis), collagen synthesis, and bacterial defense. HBOT enhances angiogenesis through a multifactorial mechanism; it stimulates fibroblast proliferation and collagen synthesis — the foundational matrix for angiogenesis. In this way, the use of adjunctive HBOT, in addition to surgical debridement and antibiotic therapy, is highly recommended. We had five cases of chronic wounds, that required a high average number of sessions (median 32.5), but the results were very positive, thus proving the benefit of this therapy. Burn wounds can also benefit from this type of treatment as they usually have a central region of coagulation bordered by a stasis zone. HBOT has been shown to reduce capillary stasis in this zone.^18,19^ We had four cases referred after burn, all with effective results after an average of 14.8 sessions. The multifactorial etiology of these patients and the absence of more objective published studies on this topic made it difficult to compare with patients who exclusively underwent conventional treatment.

Regarding hemorrhagic radical cystitis, radiotherapy induces damage to the bladder mucosa secondary to ionizing radiation, causing its atrophy, with decreased vascular and cellular density, leading to chronic hypoxia. Clinically, it can manifest as urinary symptoms such as pain and hematuria. The initial management of hemorrhagic radical cystitis is usually conservative through symptomatic treatment. However, in serious cases, HBOT has demonstrated its effectiveness and good tolerance, potentially serving as an alternative to radical cystectomy. HBOT results in a partial increase of oxygen pressure in the blood and tissues. Through repeated sessions, it stimulates neoangiogenesis and cellular mobilization of damaged areas. This reduces chronic inflammation of the bladder mucosa and leads to clinical improvement in patients.^20,21^ In our study, the majority of cases (72.4%) showed complete resolution of hematuria, and in the remaining cases, a decrease in both the severity and frequency was observed, reducing the need for Emergency Department visits. Our results are consistent with the literature, which report significant improvement following HBOT.^22,23^

Hyperbaric oxygen has also been used as an adjunctive measure in the treatment of chronic osteomyelitis, as the existing evidence suggests that hyperbaric oxygen may play a potentially advantageous role, particularly in cases of chronic osteomyelitis that are difficult to treat.^24^ Refractory osteomyelitis is often linked with compromised immune factors and prolonged antibiotic treatment or even multiple surgical interventions. When employed as a supplementary approach, HBOT along with culture-specific antibiotics and surgical debridement has shown positive outcomes. Numerous studies have suggested a favorable impact on reducing hospitalization stay when adjunctive HBOT is integrated into conventional osteomyelitis treatment protocols.^25^ In our series we had five cases of osteomyelitis referred for HBOT; all refractory to systemic antibiotic treatment and surgical intervention. They all demonstrated resolution of the condition following the sessions of HBOT, with a mean of 39.6 sessions.

HBOT has been used as adjuvant therapy for numerous sports-related injuries acquired from playing competitive sports, and there is considerable research suggesting its efficacy. HBOT accelerates recovery and enables the injured athlete to return to competition more quickly than the standard rehabilitation timeline.^26^ In our study, we have six cases referred to HBOT for sports injury, with significant improvement after a relatively low number of sessions (mean 9.5).

HBOT is generally a relatively safe therapy for various conditions; however, there are some adverse side effects. These can be divided into two categories: pressure and oxygen. The side effect of pressure is barotrauma, a condition that can affect any enclosed air-filled space, including ears, sinuses, teeth, lungs, and the bowel. The most frequent is ear barotrauma, which can be prevented or minimized by teaching auto-inflation techniques, or by inserting tympanostomy tubes, particularly in young children. The side effects of oxygen can further be subdivided into three categories: pulmonary, neurologic, and ophthalmologic.^27–29^ In our series of cases, there were just four cases of barotrauma (middle ear). Regarding the toxic effect of oxygen, this can occur at the central nervous system and pulmonary level. Central nervous system toxicity presents with symptoms such as irritability, reduced peripheral vision (tunnel vision), nausea, ringing in the ears, dizziness, muscle spasms, and widespread convulsions. Toxicity is easily reversed by interrupting the oxygen supply. At the lung level, toxicity may occur due to prolonged exposure, even at low atmospheric pressure (0.5 ATA=50% FiO_2_). The higher the partial pressure of oxygen, the shorter the time required for toxicity to emerge. Signs of pulmonary toxicity include coughing, upper airway irritation, and progressive decrease in vital capacity.^30^

In our sample, there was just one case of neurological oxygen toxicity. Presentation symptoms were irritability and nausea, which completely resolved after discontinuation of treatment. This confirms and underscores the safety of HBOT.

We acknowledge some important limitations of this study, namely its retrospective design, which limited the availability of data to collect. Additionally, patients submitted to HBOT were transferred from many different hospitals, where they had already been offered initial treatments. Therefore, we could not control or gather data, which may have had different impacts on patients’ outcomes. Furthermore, the number of sessions varied widely among patients, due to the inexistence of validated pediatric protocols for HBOT in Portugal, and it could be questioned the true therapeutic contribution to each case.

Apart from these limitations, to our knowledge, this is the first Portuguese study assessing the management of HBOT in pediatric patients, and it can contribute to characterizing the impact of this modality of treatment on the pediatric population.

Pediatricians are not always aware of the potential benefits that HBOT can offer in recommended situations. However, some aspects must be considered before starting treatment with hyperbaric oxygen in a child. A close cooperation between pediatricians and hyperbaric medicine teams is very important to obtain optimal results with this therapy.

## Data Availability

The database that originated the article is available with the corresponding author.
